# *Methylenetetrahydrofolate reductase* C677T (Ala>Val, rs1801133 C>T) polymorphism decreases the susceptibility of hepatocellular carcinoma: a meta-analysis involving 12,628 subjects

**DOI:** 10.1042/BSR20194229

**Published:** 2020-02-20

**Authors:** Sheng Zhang, Jiakai Jiang, Weifeng Tang, Longgen Liu

**Affiliations:** 1 Department of General Surgery, Changzhou No. 3 People’s Hospital, Changzhou, Jiangsu Province, China; 2 Department of Cardiothoracic Surgery, Affiliated People’s Hospital of Jiangsu University, Zhenjiang, Jiangsu Province, China; 3 Department of Liver Disease, Changzhou No. 3 People’s Hospital, Changzhou, Jiangsu Province, China

**Keywords:** Hepatocellular carcinoma, Meta-analysis, MTHFR, Polymorphism, Susceptibility

## Abstract

C677T (Ala>Val, rs1801133 C>T), a non-synonymous variant of *methylenetetrahydrofolate reductase* (*MTHFR*) gene, has been found to be associated with an impair enzyme activity of MTHFR. The relationship of *MTHFR* rs1801133 with hepatocellular carcinoma (HCC) has been extensively investigated. However, the findings were conflicting. Recently, more investigations have been conducted on the relationship of *MTHFR* rs1801133 with HCC. To obtain a more precise assessment on the effect of this non-synonymous variant to the development of HCC, a pooled-analysis was performed. This meta-analysis consisted of 19 independent case–control studies. By using the odds ratio (OR) combined with 95% confidence interval (CI), the relationship of *MTHFR* rs1801133 with HCC risk was determined. A total of 19 independent case–control studies were included. Finally, 6,102 HCC cases and 6,526 controls were recruited to examine the relationship of *MTHFR* rs1801133 with HCC risk. In recessive model (TT vs. CC/CT), the findings reached statistical significance (OR, 0.90; 95%CI, 0.82–0.98; *P* = 0.016). Subgroup analysis also found an association between *MTHFR* rs1801133 polymorphism and the decreased risk of HCC in hepatitis/virus related patients (recessive model: OR, 0.85; 95%CI, 0.72–0.99; *P* = 0.035, and allele model: OR, 0.90; 95%CI, 0.81–0.99; *P* = 0.028). Subgroup analyses indicated that extreme heterogeneity existed in Asian population, larger sample size investigation, hospital-based study and normal/healthy control subgroups. The shape of Begger’s seemed symmetrical. Egger’s linear regression test also confirmed these evaluations. Sensitivity analyses suggested that our findings were stable. In summary, our results highlight that *MTHFR* rs1801133 polymorphism decreases HCC susceptibility. The relationship warrants a further assessment.

## Introduction

In 2018, global cancer statistics estimated that liver malignancy was the fifth most frequent type of cancer incidence among men and the eleven most frequent type among women, about 596,574 and 244,506 new cases diagnosed worldwide, respectively [1]. However, the fatality was the third most frequent type [1]. The etiology of liver cancer (LC) was not well-established. Hepatocellular carcinoma (HCC) is one of the most important primary LC, which comprised almost 80% of LC cases. Some major susceptibility factors (e.g. aflatoxin-contaminated food, superabundant drinking, tobacco consumption, chronic virus infection, higher body mass index and Type 2 diabetes) [2–6] may contribute to the development of HCC. Additionally, hereditary factor has also been suggested to affect the susceptibility for the occurrence of HCC.

*Methylenetetrahydrofolate reductase* (*MTHFR*) locates in 1p36.3, which maps from 11785723 to 11806103 (GRCh38; April, 2018). MTHFR, a key enzyme, plays a vital effect in folate metabolism by the role of catalyzing the 5,10-methylenetetrahydrofolate (5,10-methylene-THF) to 5-methyltetrahydrofolate (5-methylene-THF) irreversibly. In the conversion of homocysteine to methionine, 5-methylene-THF is a primary methyl donor [7]. *MTHFR* rs1801133 (C677T), a non-synonymous variant (Ala>Val), has been suggested to influence the activity of MTHFR enzyme [8]. The correlation of *MTHFR* rs1801133 polymorphism with malignancy has been extensively explored. This single-nucleotide polymorphism (SNP) was suggested to be associated with thyroid cancer [9], colorectal cancer [10,11], breast cancer [12], esophagogastric junction adenocarcinoma [13], non-small cell lung cancer [14], acute lymphoblastic leukemia [15,16], gastric cancer [17], renal cell carcinoma [18] and esophageal carcinoma [19], among others.

Recently, many case–control studies have been carried out to determine the relationship of *MTHFR* rs1801133 polymorphism with the development of HCC [19–29]. However, the observations were controversial. Several meta-analyses also got conflicting results. To shed light on this issue, we conducted an extensive pooled-analysis to determine the role of *MTHFR* rs1801133 polymorphism on the development of HCC.

## Materials and methods

### Study searching

Publications were obtained by searching the PubMed and EMBASE databases before October 19, 2019. The following strategy was used: (Methylenetetrahyfrofolate reductase OR MTHFR OR rs1801133) AND (SNP OR polymorphism) AND (cancer OR carcinoma) and (hepatocellular OR liver). The references in reviews and meta-analyses were also retrieved to get data. In this pooled-analysis, there was no language limited.

### Inclusion criteria

In our meta-analysis, the eligible criteria of the included publications were: (1) designed as a case–control study; (2) focusing on the relationship of the *MTHFR* rs1801133 polymorphism with HCC risk; (3) genotype data could be extracted and (4) publications were compatible with Hardy–Weinberg equilibrium (HWE) in controls.

### Exclusion criteria

The criteria for exclusion were as following: (1) publications incompatible with HWE; (2) overlapping data; (3) not case–control study design and (4) only focusing on the relationship of *MTHFR* rs1801133 polymorphism with HCC survival.

### Data extraction

The authors (S. Zhang and J. Jiang) extracted the following data: the surname of first author, publication year, populations studied, country where the investigation was carried out, ratio of sex, age, drinking, positive (%) of hepatitis B surface antigen (HBsAg), genotyping method, the number of participants and *MTHFR* rs1801133 genotype. If there was conflicting assessment, another reviewer (W. Tang) was invited. During this process, they made a vote to obtain the final decision.

### Statistical methods

In the present study, the odds ratios (ORs) combined with 95% confidence intervals (CIs) were harnessed to compare the difference between HCC group and controls. *P* value (<0.05) was considered statistically significant. The present meta-analysis determined the correlation in four genetic models [e.g. dominant model (TT/CT vs. CC), homozygote model (TT vs. CC), allele model (T vs. C) and recessive model (TT vs. CC/CT)]. Using *I^2^* metric and Q statistic, the heterogeneity among the eligible case–control studies was evaluated. If *P* < 0.10 or *I*^2^ > 50%, we defined that there was significant heterogeneity. Thus, the random-effect model was used [30,31]. Otherwise, there was no heterogeneity detected. A fixed-effect model was used to combine the data [32]. The Egger test and Begg’s test were used to assess the bias of publication. If *P* < 0.10, we defined that there was a significant publication bias. By omitting a study one by one and analyzing the remainders, sensitivity analysis was performed to assess the stability of our findings. The distribution of the *MTHFR* rs1801133 genotype was used to calculate the *P* value of HWE by using an online software (http://ihg.gsf.de/cgi-bin/hw/hwa1.pl) in controls [33–35]. STATA 12.0 software (Stata Corp., College Station, Texas) was used to conduct the analysis. In this study, *P* value was two sided.

### Quality assessment of meta-analysis

Two authors (S.Z. and J.J.) independently extracted the data and calculated the quality score of the included case–control studies. The detailed scores were determined by a quality assessment criteria, which were presented in previous studies [36,37]. If the scores were more than 6.0, the investigation had an acceptable quality [38].

## Results

### Eligible studies

**Figure 1:**
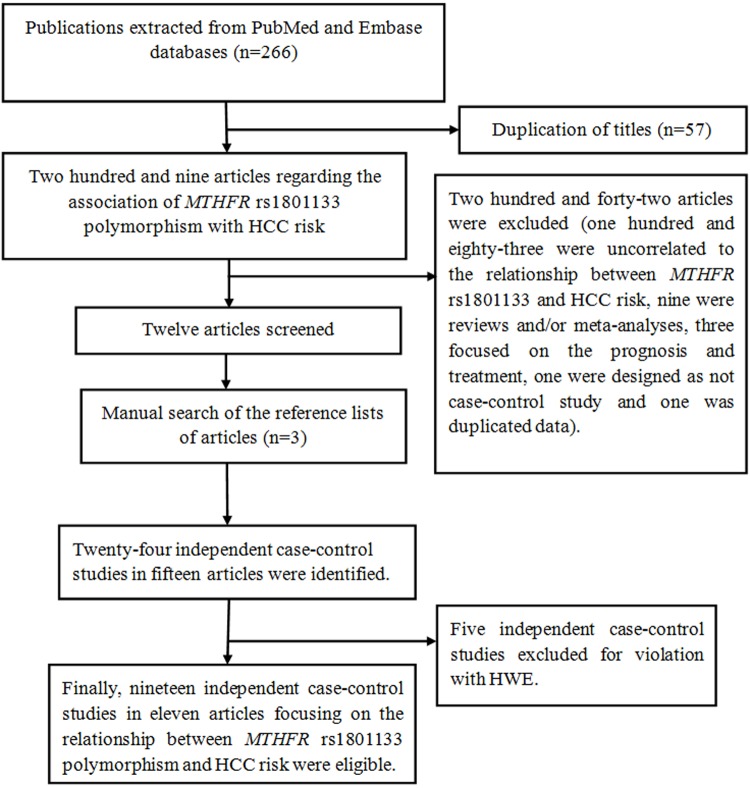
Flow diagram of this meta-analysis

A total of 11 publications were eligible (Figure 1). Four articles involved several different subgroups, so we considered them as independent investigations. After a screening, 19 independent case–control studies were included. In addition, five publications were excluded for incompatible with HWE [29,39,40–42]. Finally, 6,102 HCC cases and 6,526 controls were recruited to examine the relationship of *MTHFR* rs1801133 polymorphism with HCC risk (Table 1). The publication year covered from 2004 to 2019. These investigations were performed in different populations: three were conducted in mixed populations [28,29], four were carried out in Caucasians [26,27], and twelve involved Asians [19–25]. The *MTHFR* rs1801133 genotypes are summarized in Table 2.

**Table 1:** Characteristics of the studies in meta-analysis

Study	Year	Sample size	Country	Ethnicity	Sex, male (%); Case/Control	Age (years); Case/Control	Drinkinge (%); Case/Control	HBsAg, positive (%); Case/Control	Genotype method	Source of control	Type of control
Cui	2012	356/641	China	Asian	83.1/43.5	56.6/58.7	44.1/30.3	77.5/8.6	Real-time PCR	PB	Normal or healthy control
Fabris	2009	65/147	Italy	Caucasian	NA/NA	NA/NA	NA	26.2/10.9	PCR-RFLP	HB	Hepatitis or virus related control
Fabris	2009	65/236	Italy	Caucasian	NA/69.5	NA/48	NA	NA/NA	PCR-RFLP	HB	NA
Jiao	2017	726/549	China	Asian	72.7/54.6	56.5/41.5	24.5/NA	89.1/0.0	TaqMan	HB	Normal or healthy control
Jiao	2017	726/558	China	Asian	72.7/53.6	56.5/33.7	24.5/NA	89.1/0.0	TaqMan	HB	Normal or healthy control
Jiao	2017	726/81	China	Asian	72.7/61.7	56.5/34.4	24.5/NA	89.1/100	TaqMan	HB	Hepatitis or virus related control
Jiao	2017	726/442	China	Asian	72.7/66.5	56.5/39.6	24.5/13.6	89.1/100	TaqMan	HB	Hepatitis or virus related control
Jiao	2017	726/704	China	Asian	72.7/64.9	56.5/53.7	24.5/23.6	89.1/88.7	TaqMan	HB	Hepatitis or virus related control
Kwak	2008	96/201	Korea	Asian	NA	57.6/53.6	NA	NA	PCR-RFLP	HB	Normal or healthy control
Peres	2016	71/356	Brazil	Mixed	73.2/73.3	NA	62.0/46.0	NA	PCR-RFLP	HB	Normal or healthy control
Peres	2016	71/116	Brazil	Mixed	73.2/74.1	NA	62.0/53.4	NA	PCR-RFLP	HB	Hepatitis or virus related control
Saffroy	2004	72/122	France	Caucasian	84.7/85.2	55/50	NA	NA	PCR-RFLP	HB	Hepatitis or virus related control
Saffroy	2004	27/80	France	Caucasian	74.1/86.3	54/54	NA	NA	PCR-RFLP	HB	Normal or healthy control
Saffroy	2004	49/30	France	Caucasian	85.7/66.7	56/52	NA	NA	PCR-RFLP	HB	Hepatitis or virus related control
Xu	2014	205/200	China	Asian	NA	52.0/61.0	NA	NA	PCR	NA	NA
Yuan	2007	118/209	USA	Mixed	68.6/61.2	NA	71.2/68.4	28.0/11.5	TaqMan	PB	Normal or healthy control
Zhu	2006	508/543	China	Asian	85.8/48.8	50/45	39.8/17.9	72.8/17.9	PCR-RFLP	HB	Normal or healthy control
Chang	2014	204/415	China	Asian	77.9/69.2	53.9/57.7	41.7/35.7	64.7/24.6	PCR-RFLP	PB	Normal or healthy control
Zhang	2019	584/923	China	Asian	89.9/90.5	53.2/53.7	29.1/16.0	70.6/9.2	SNPscan	HB	Normal or healthy control

PCR-RFLP: polymerase chain reaction-restriction fragment length polymorphism

PCR: polymerase chain reaction

SNP: single-nucleotide polymorphism

HP: hospital-based

PB: population-based

NA: not available

**Table 2:** Distribution of *MTHFR* rs1801133 C>T polymorphism genotype and allele

Study	Year	Case TT	Case CT	Casde CC	Control TT	Control TC	Control CC	Case T	Case C	Control T	Control C	HWE	Quality assessment
Cui	2012	125	179	52	195	325	121	429	283	715	567	Yes	7.0
Fabris	2009	13	–	CC/CT = 52	23	–	CC/CT = 124	–	–	–	–	Yes	6.5
Fabris	2009	13	–	CC/CT = 52	54	113	69	–	–	–	–	Yes	6.5
Jiao	2017	188	370	168	176	263	110	746	706	615	483	Yes	7.5
Jiao	2017	188	370	168	169	268	121	746	706	606	510	Yes	7.5
Jiao	2017	188	370	168	29	35	17	746	706	93	69	Yes	6.5
Jiao	2017	188	370	168	120	222	100	746	706	462	422	Yes	7.5
Jiao	2017	188	370	168	215	338	151	746	706	768	640	Yes	7.5
Kwak	2008	18	46	32	31	106	64	82	110	168	234	Yes	6.5
Peres	2016	7	36	28	33	174	149	50	92	240	472	Yes	7.0
Peres	2016	7	36	28	13	55	48	50	92	81	151	Yes	6.0
Saffroy	2004	5	24	43	10	60	52	34	110	80	164	Yes	6.5
Saffroy	2004	2	16	9	13	37	30	20	34	63	97	Yes	6.5
Saffroy	2004	5	29	15	3	17	10	39	59	23	37	Yes	6.5
Xu	2014	50	112	43	50	111	39	212	198	211	189	Yes	6.5
Yuan	2007	14	51	53	30	99	80	79	157	159	259	Yes	7.0
Zhu	2006	110	226	172	102	268	173	446	570	472	614	Yes	8.0
Chang	2014	30	114	50	57	199	135	174	214	313	469	Yes	7.5
Zhang	2019	49	227	299	103	446	372	325	825	652	1190	Yes	8.0

HWE: Hardy–Weinberg equilibrium.

### Meta-analysis results

In the eligible investigations, the MAF of *MTHFR* rs1801133 C/T polymorphism was 0.475 in HCC patients (5,670/11,944) and was 0.466 in controls (5,721/12,288). In different race, the MAF of controls was not similar in controls. The MAFs were 0.352 (480/1,362) in mixed populations, 0.328 (214/652) in Caucasians, and that was 0.485 (5,075/10,462) in Asians.

**Figure 2:**
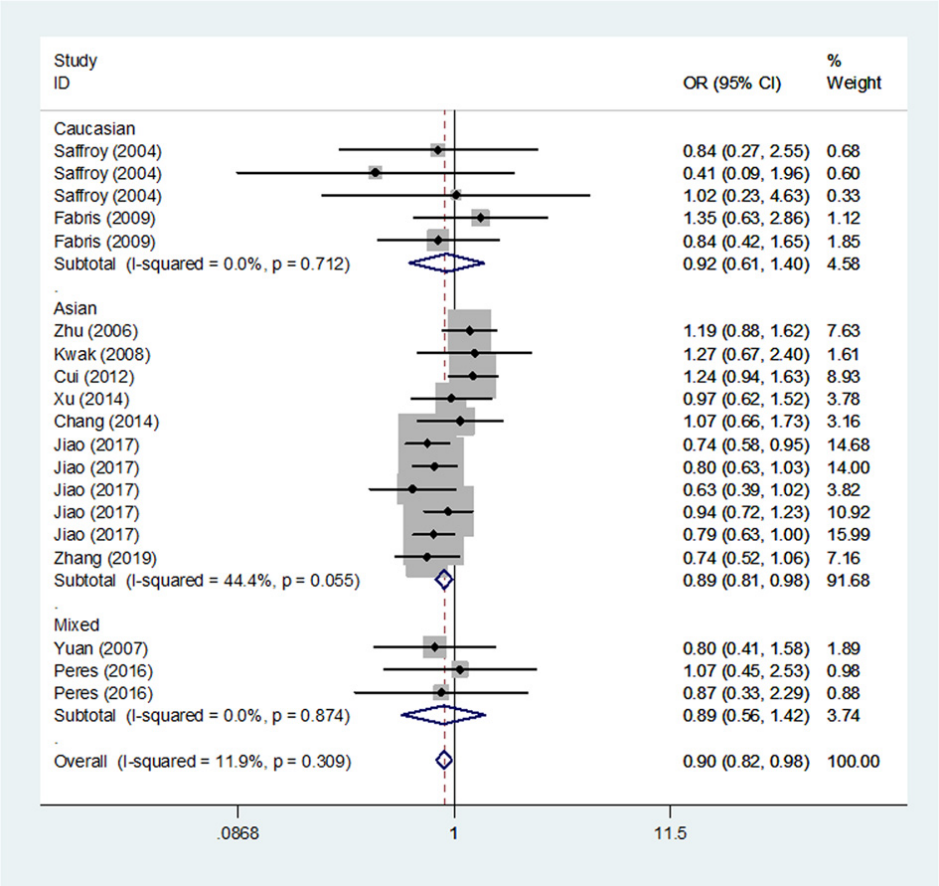
Meta-analysis of the association between *MTHFR* rs1801133 polymorphism and HCC risk (recessive model, fixed-effects model)

The pooled-analysis findings were reported in four genetic models including 19 independent case–control studies. In recessive model (TT vs. CC/CT), the findings reached statistical significance (OR, 0.90; 95%CI, 0.82–0.98; *P* = 0.016, Table 3 and Figure 2). In other genetic models, we failed to obtain the significance (dominant model: OR, 0.92; 95%CI, 0.81–1.05; *P* = 0.209, homozygote model: OR, 0.88; 95%CI, 0.77–1.01; *P* = 0.078, and allele model: OR, 0.93; 95%CI, 0.85–1.01; *P* = 0.077, Table 3).

**Table 3:** Results of the meta-analysis from different comparative genetic models

	No. of studies	T vs. C				TT vs. CC				TT/CT vs. CC				TT vs. CT/CC			
		OR (95% CI)	*P*	*I^2^*	*P*(Q-test)	OR(95% CI)	*P*	*I^2^*	*P*(Q-test)	OR(95% CI)	*P*	*I^2^*	*P*(Q-test)	OR(95% CI)	*P*	*I^2^*	*P*(Q-test)
Total	19	0.93(0.85–1.01)	0.077	48.7%	0.013	0.88(0.77–1.01)	0.078	25.8%	0.158	0.92(0.81–1.05)	0.209	47.3%	0.016	**0.90(0.82–0.98)**	**0.016**	11.9%	0.309
Ethnicity																	
Asians	11	0.94(0.85–1.03)	0.182	63.4%	0.002	0.90(0.75–1.06)	0.210	50.1%	0.029	0.94(0.80–1.09)	0.380	59.3%	0.006	0.90(0.79–1.03)	0.133	44.4%	0.055
Caucasians	5	0.79(0.57–1.09)	0.153	0.0%	0.405	0.67(0.30–1.50)	0.331	0.0%	0.781	0.73(0.47–1.13)	0.155	42.2%	0.177	0.92(0.61–1.40)	0.693	0.0%	0.712
Mixed	3	0.94(0.76–1.17)	0.592	0.0%	0.549	0.86(0.52–1.41)	0.550	0.0%	0.721	0.94(0.70–1.27)	0.679	0.0%	0.494	0.89(0.56–1.42)	0.619	0.0%	0.874
Sample sizes																	
<1000	13	1.02(0.93–1.13)	0.631	25.8%	0.198	1.06(0.86–1.31)	0.559	0.0%	0.488	1.05(0.90–1.22)	0.537	31.3%	0.149	1.02(0.87–1.19)	0.857	0.0%	0.686
≥1000	6	**0.88(0.80–0.96)**	**0.006**	52.6%	0.061	**0.80(0.70–0.92)**	**0.001**	29.1%	0.217	**0.84(0.73–0.97)**	**0.020**	48.8%	0.082	**0.85(0.76–0.94)**	**0.002**	35.3%	0.172
Source of control																	
P-B	3	1.10(0.89–1.36)	0.374	53.7%	0.116	1.30(0.97–1.73)	0.080	39.9%	0.190	1.19(0.81–1.75)	0.385	65.0%	0.057	1.14(0.91–1.43)	0.248	0.0%	0.490
H-B	15	**0.88(0.83–0.93)**	**<0.001**	22.9%	0.212	**0.81(0.71–0.92)**	**0.001**	0.0%	0.642	**0.84(0.77–0.93)**	**<0.001**	23.2%	0.209	**0.85(0.77–0.94)**	**0.002**	0.0%	0.498
NA	1	0.96(0.73–1.26)	0.766	–	–	0.91(0.51–1.63)	0.743	–	–	0.91(0.56–1.48)	0.712	–	–	0.97(0.62–1.52)	0.887	–	–
Control type																	
Normal or healthy	10	0.95(0.84–1.08)	0.428	66.3%	0.002	0.92(0.74–1.16)	0.487	53.9%	0.021	0.95(0.79–1.15)	0.590	65.0%	0.002	0.94(0.79–1.11)	0.439	42.3%	0.076
Hepatitis or virus related	7	**0.90(0.81–0.99)**	**0.028**	0.0%	0.533	0.82(0.67–1.00)	0.054	0.0%	0.905	0.90(0.56–1.06)	0.208	0.0%	0.463	**0.85(0.72–0.99)**	**0.035**	0.0%	0.695
NA	2	0.96(0.73–01.26)	0.766	–	–	0.91(0.51–1.63)	0.743	–	–	0.91(0.56–1.48)	0.712	–	–	0.93(0.64–1.35)	0.684	0.0%	0.729

P-B: population-based;

H-B: hospital-based

NA: not available

Subgroup analyses were carried out according to the following terms: ethnicity (Caucasians or Asians or mixed), sample sizes (<1000 or ≥1000 subjects), control type [normal/healthy subjects or hepatitis/virus related patients or not available (NA)] and source of control [hospital-based (HB) or population-based (PB) or NA]. We pooled seven case–control studies (including 2,435 HCC cases and 1,642 hepatitis/virus related patients) and found an association between *MTHFR* rs1801133 polymorphism and decreased risk of HCC in hepatitis/virus related patients (recessive model: OR, 0.85; 95%CI, 0.72–0.99; *P* = 0.035, and allele model: OR, 0.90; 95%CI, 0.81–0.99; *P* = 0.028, Table 3). When we conducted a subgroup analysis by ethnicity, null association between *MTHFR* rs1801133 C>T polymorphism and the risk of HCC was found.

### Heterogeneity assessment

In some genetic models, heterogeneity was significant (Table 3). Subgroup analyses indicated that extreme heterogeneity existed in Asian populations, larger sample size investigation, HB study and normal/healthy control subgroups. If we excluded these subgroups in our meta-analysis, the heterogeneity significantly decreased.

### Bias evaluation

**Figure 3:**
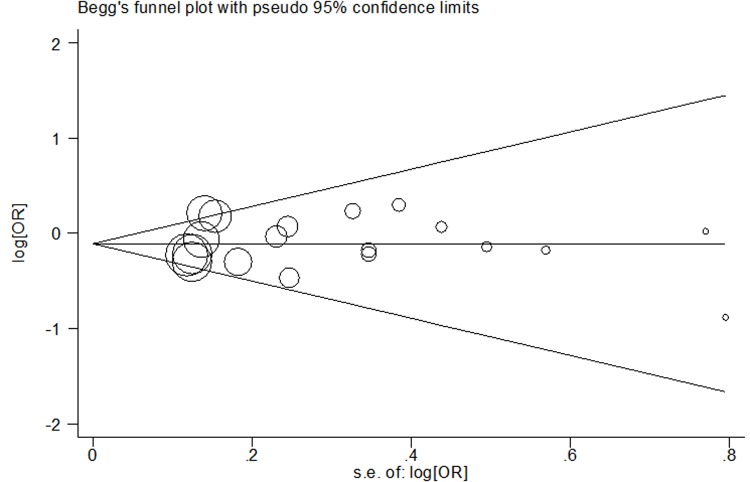
Begg’s funnel plot of meta–analysis of the association between *MTHFR* rs1801133 polymorphism and HCC risk (recessive model, fixed-effects model)

We used Begger’s and Egger’s tests to identify the bias of publication among the included investigations. The shape of Begger’s test seemed symmetrical (Figure 3). Egger’s linear regression test also confirmed these evaluations.

### Sensitivity analyses

**Figure 4:**
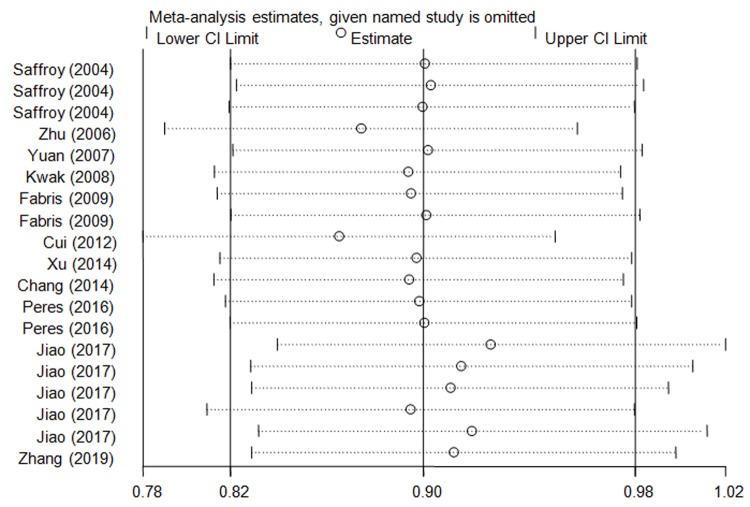
Sensitivity analysis of the influence of *MTHFR* rs1801133 polymorphism to HCC risk (recessive model, fixed-effects model)

By sequentially omitting an individual investigation, sensitivity analysis was carried out. This method is considered as a criterion for meta-analysis. The results indicated that the significance of the present study could not be altered by removing any case–control study (Figure 4), suggesting that our findings were stable.

### Quality assessment

Table 2 presents the results of the quality evaluation. Each eligible study had an acceptable quality (scores ≥ 6).

## Discussion

Accumulating investigations highlight that *MTHFR* rs1801133 polymorphism may be associated with the development of HCC. However, the findings of the previous case–control studies were conflicting, with several investigations suggesting a potential relationship, whereas others did not support the correlation. In this investigation, to explore whether the *MTHFR* rs1801133 polymorphism was implicated in the etiology of HCC, we carried out a pooled-analysis of 19 eligible studies, which recruited 6,102 HCC cases and 6,526 controls. This meta-analysis indicated that the *MTHFR* rs1801133 polymorphism was a protective factor for the development of HCC in the overall comparison. Compared with the previous study, this pooled-analysis first confirmed the association of *MTHFR* rs1801133 polymorphism with a decreased risk of HCC.

*MTHFR* rs1801133 polymorphism locates on 11796321 (NCBI Build 38) of Chromosome 1. Zhu and her/his colleagues first reported that *MTHFR* rs1801133 polymorphism might confer a risk to HCC [24]. In addition, Cui et al. also suggested that this polymorphism could increase the risk of HCC [20]. However, some case–control studies indicated that the rs1801133 polymorphism in *MTHFR* gene might decrease the susceptibility of HCC [21,25]. And most studies reported that this SNP in *MTHFR* gene could not alter the risk of HCC. Thus, the association of *MTHFR* rs1801133 polymorphism with the susceptibility of HCC was more conflicting. Here, we performed a pooled-analysis of nineteen eligible studies involving 6,102 HCC cases and 6,526 controls to explore the correlation of rs1801133 with the etiology of HCC. The results indicated that this SNP in *MTHFR* gene could be a protective factor for the occurrence of HCC. Two meta-analyses suggested that rs1801133 was not associated with HCC development [43,44]. Others pooled-analyses reported that *MTHFR* rs1801133 polymorphism was associated with an increased risk of HCC [45–48]. Compared with these early meta-analyses, our analysis included more large sample size studies [21,25]. It is worth mentioning that these more recent case–control studies have recruited more participants and reported that rs1801133 polymorphism was a protective factor for the development of HCC. Compared with the most recent meta-analysis [23], the merit of our study was the larger sample size and the detailed subgroup analysis. Combined the eligible studies, we observed that rs1801133 decreased the susceptibility of HCC in the overall comparison. The quality score was evaluated in our study. Each eligible study had an acceptable quality (scores ≥6). This indicated that our findings were reliable. We also found an association between *MTHFR* rs1801133 polymorphism and decreased risk of HCC in hepatitis/virus related patients. Of late, in Asian population, some meta-analyses identified that *MTHFR* rs1801133 polymorphism decreased the risk of colorectal cancer [49,50]. Some publications [51,52] suggested that *MTHFR* rs1801133 C>T polymorphism (Ala→Val) could promote the level of 5,10-methylene-THF for DNA synthesis, which might be protective to carcinogenesis. In the future, a functional study should be carried out to address how this Ala→Val substitution could decrease the risk of HCC.

Heterogeneity was identified in the overall comparison. In the present study, we conducted subgroup analyses to explore the major source among the eligible studies. Subgroup analysis suggested that major heterogeneity might be due to different populations, sample size, and characteristics of controls.

Some potential limitations should be addressed in this pooled-analysis. First, only published investigations were eligible in our study. Thus, the number of included case–control studies might be inadequate. Second, for lacking of sufficient data, only crude ORs and CIs were calculated. Third, the controls in some of the case–control studies were hepatitis or virus related patients. Fourth, a recent investigation contained some subgroups, we treated them as independent case–control studies. However, in this literature, the same HCC group was used in different stratified analysis. Finally, our study did not focus on the gene–gene and gene–environment interactions.

In summary, the present pooled-analysis highlights that *MTHFR* rs1801133 polymorphism is a protective factor for the occurrence of HCC, especially in hepatitis/virus related patients. The relationship of *MTHFR* rs1801133 polymorphism with HCC risk warrants a further determination.

## Abbreviations

CI: confidence interval
HCC: hepatocellular carcinoma
*MTHFR*: *methylenetetrahydrofolate reductase*
OR: odds ratio
